# Evaluating the mechanisms and long-term effects of a web-based comprehensive sexual health and media literacy education program for young adults attending community college: study protocol for a three-arm randomized controlled trial

**DOI:** 10.1186/s13063-022-06414-6

**Published:** 2022-06-21

**Authors:** Tracy M. Scull, Christina V. Dodson, Reina Evans-Paulson, Liz C. Reeder, Jacob Geller, Kathryn N. Stump, Janis B. Kupersmidt

**Affiliations:** Innovation Research & Training, 5316 Highgate Drive, Suite 125, Durham, NC 27713 USA

**Keywords:** Sexual health, Sexual violence prevention, Media literacy education, Community college, Web-based learning, Adolescent health

## Abstract

**Background:**

Many community college students experience poor sexual and relationship health outcomes. Young adults consume a plethora of media content, much of which depicts unhealthy sexual and romantic relationships, and research has shown that media exposure can negatively impact health outcomes. Asynchronous, web-based media literacy education (MLE) programs have been shown to improve short-term sexual and relationship health outcomes. However, there is a dearth of research on the mechanisms by which MLE programs impact health outcomes and the long-term effects of MLE programs on sexual and relationship health outcomes among community college students.

**Methods:**

This study will (1) evaluate the unique effects of MLE on primary and secondary sexual and relationship health outcomes; (2) compare the mechanisms underlying the effects of an asynchronous, web-based MLE sexual health program (*Media Aware*) to the mechanisms underlying the effects of an active control program on health outcomes; and (3) evaluate the long-term efficacy of *Media Aware* on media literacy skills and sexual and relationship health outcomes compared to active control and delayed intervention control groups. To address these aims, a three-arm randomized controlled trial with young adults attending community college will be conducted. It is expected that around 30 campuses will participate and approximately 67 students from each campus will be enrolled in the study (estimated *n* = 2010). Campuses will be randomized to either the (1) intervention group (*Media Aware*); (2) active control group (sexual health education from *Media Aware* without MLE content or methods); or (3) delayed intervention control group. Students will complete online questionnaires at pretest, posttest, 6-month, and 12-month follow-ups.

**Discussion:**

This project has the potential to advance theory about the potential mechanisms through which MLE has an impact on sexual and relationship health outcomes by directly testing the impact of interventions using a randomized design. Additionally, this study is expected to establish strong evidence for the effectiveness of *Media Aware* for use with young adults and to help identify strategies to optimize the longer-term impact of the program on health. Students’ satisfaction with programming will be discussed to inform future implementation efforts.

## Administrative information

Note: the numbers in curly brackets in this protocol refer to SPIRIT checklist item numbers. The order of the items has been modified to group similar items (see http://www.equator-network.org/reporting-guidelines/spirit-2013-statement-defining-standard-protocol-items-for-clinical-trials/).

**Table:** 

**Title {1}**	Evaluating the mechanisms and long-term effects of a web-based comprehensive sexual health and media literacy education program for young adults attending community college: Study protocol for a three-arm randomized controlled trial.
**Trial registration {2a and 2b}**	ClinicalTrials.gov, NCT04950686.
**Protocol version {3}**	This is the first version of the protocol. The ClinicalTrials.gov version identifier is CCHealthStudy-R01-20-007 (registered on 3 May 2021).
**Funding {4}**	This study was funded by the Eunice Kennedy Shriver National Institute of Child Health and Human Development of the National Institutes of Health (NIH) under award number R01HD099134 to TS. Research reported in this paper is solely the responsibility of the authors and does not necessarily represent the official views of the NIH. Aside from monitoring regular reports, it is not anticipated that NIH will be further involved in the design of the study, data collection, analysis, and interpretation, or in writing the manuscript.
**Author details {5a}**	TMS received her Ph.D. in Developmental Psychology from Duke University and is now a Senior Research Scientist at innovation Research & Training. CVD received her Ph.D. in Mass Communication from the University of North Carolina at Chapel Hill and is now a Research Scientist at innovation Research & Training. REP received her Ph.D. in Applied Social and Community Psychology from North Carolina State University and is now a Research Scientist at innovation Research & Training. LCR received her B.S. in Psychology from the University of North Carolina at Chapel Hill and is now a Research Assistant at innovation Research & Training. JG received his B.A. in Sociology from the University of North Carolina at Chapel Hill and worked as a Research Assistant at innovation Research & Training. KNS received her Ph.D. in Developmental Psychology from University of Kansas and is now a Research Scientist at innovation Research & Training. JBK received her Ph.D. in Clinical Psychology from Duke University and is now a Senior Research Scientist at innovation Research & Training.
**Name and contact information for the trial sponsor {5b}**	innovation Research & Training 5316 Highgate Drive, Suite 125 Durham, NC 27713 919.493.7700
**Role of sponsor {5c}**	The investigators at innovation Research & Training are responsible for the design of the study, data collection, analysis, interpretation of results, dissemination, and other aspects of the day-to-day study operations.

## Background and rationale {6a}

Many community college students experience poor sexual and relationship health outcomes which can impact their lifelong well-being. Community college students are at greater risk than their 4-year college peers for unintended pregnancy, sexually transmitted infections (STIs), such as HIV, and dating violence [1–4]. Importantly, young adults (ages 18–19) in this group are in a developmental stage marked by rapid neurobiological development and susceptibility to peer pressure—factors which may leave them at even greater risk [5]. Research also shows that media can impact young adults’ health outcomes. Media often normalize and glamorize unhealthy sexual and romantic relationships [6]. Exposure to sexual media content has been linked to initiation of sexual activity, teen pregnancy, endorsement of gender stereotypes, and acceptance of dating violence [7–9]. In sum, it is essential that young adults attending community college receive educational experiences and resources that are evidence-based and that are designed to promote sexual and relationship health, as well as prevent sexual violence. Furthermore, more comprehensive sexual education programs that also address the role that media play in shaping and reinforcing unhealthy sexual and relationship health norms, attitudes, and behaviors may be especially impactful.

*Media Aware for Young Adults* (i.e., *Media Aware*) is an evidence-based comprehensive sexual health education program that was developed to address these needs by providing young adults with the knowledge and skills essential for critically analyzing media messages and making healthy sexual and relationship decisions [10]. *Media Aware* is grounded in media literacy education (MLE) and theory. Media literacy is broadly defined as “the ability to access, analyze, evaluate, create and act using all forms of communication” [11]. MLE aims to enhance critical thinking about media messages related to health behaviors (e.g., Is it realistic that those characters had unprotected sex with no consequences?) as well as provide missing information about health that is frequently left out of media messages (e.g., using latex condoms can prevent unplanned pregnancy and the spread of STIs). MLE has been found to be an effective approach to sexual health promotion [10, 12–14].

A short-term evaluation of *Media Aware* found that the program led to improved media- and health-related outcomes, including, a reduction in risky sexual behaviors among community college students aged 18–19 [10]. Despite these positive findings, a number of gaps in the literature were identified. First, MLE is a relatively new approach to sexual health promotion, and while evidence supports its effectiveness, there are no studies that have isolated the effects of MLE (i.e., enhancing critical thinking about media messages) in sexual health promotion to determine the independent effects of using MLE in sexual health programming over and above sexual health programming that does not include MLE. Second, research is needed to better understand the mechanisms underlying MLE’s impact on sexual health outcomes and explore how these effects may differ from the mechanisms underlying the effects of sexual health programming that does not include MLE. Understanding the mediators of behavioral effects would advance theory and inform the development of other theory-based MLE prevention programs*.* Third, while a previous study established *Media Aware*’s immediate positive effects on young adults’ media processing and sexual health outcomes, additional research is needed to determine if the program’s effects are sustained over time.

## Objectives {7}

This study has three objectives designed to address three critical gaps in the literature on MLE and sexual health promotion. The first objective is to evaluate the immediate short-term effects of MLE on media-related outcomes (i.e., media literacy skills), sexual and relationship health cognitions (e.g., sexual health beliefs, intentions, and attitudes), and sexual and relationship health behaviors (e.g., sexual communication, dating violence, condom use) compared to the active control and delayed intervention control groups. The second objective is to compare the mechanisms underlying *Media Aware*’s effects on health outcomes to the mechanisms underlying the effects of an active control program. The third objective is to evaluate the long-term efficacy of *Media Aware* on media-related outcomes and sexual and relationship health outcomes compared to the active control and delayed intervention control groups.

## Trial design {8}

To address these objectives, we will conduct a three-arm randomized controlled superiority trial with young adults (i.e., ages 18–19) attending community college. It is expected that about 30 campuses will participate and approximately 67 students from each campus will be enrolled (estimated *n* = 2010) in the study. Community college campuses will be randomized to either the (1) intervention group (receives *Media Aware*); (2) active control group (receives *Health Aware*: a program with sexual health information but no MLE content); or (3) delayed intervention control group (receives no sexual health education program until the end of the study). Students from each campus will complete web-based questionnaires at pretest, posttest, 6-month, and 12-month follow-ups. Participants and outcome assessor will be masked: participants will not be informed which conditions are considered intervention versus control and all measures are assessed using a web-based data collection system rather than a human outcomes assessor.

## Methods

### Study setting {9}

Participants will be recruited from community college campuses located across the USA that are diverse with respect to their geographic location (e.g., rurality) as well as the racial-ethnic make-up of the student body. The program as well as the study questionnaires can be completed using a smartphone, tablet, or computer.

### Eligibility criteria {10}

For students to be eligible to participate in the study, the following inclusion criteria must be met: (1) participant must be an 18- or 19-year-old community college student; (2) participants must be enrolled at one of the community college campuses from which we are recruiting for the study; (3) students must have an email address in order to receive communications about the study and must have access to a computer, tablet, or smartphone device with internet access. In addition, the study materials (e.g., questionnaires, programs) are in English; therefore, students must be able to speak and read English. Finally, no high school students who are dual enrolled in high school as well as community college classes will be eligible to participate in the study. Quotas will be used to guide enrollment and ensure that a diverse student sample with respect to race, ethnicity, and gender is recruited. Therefore, not all students who meet the eligibility criteria will be enrolled in the study.

### Who will take informed consent? {26a}

Interested students will complete a brief eligibility survey that is located on the study recruitment website. Following completion of the web-based eligibility survey, potentially eligible students will be asked to review and endorse an online consent form which outlines the objectives of the study, describes the measures and procedure included for involvement as a participant in the project, and details the potential risks and benefits of the research. The consent form will include contact information for the PI, project director, and IRB chair, so that potential participants may contact them to ask any questions they may have about the study. Consent forms will state that participants may discontinue their participation at any time for any reason without consequences.

### Additional consent provisions for collection and use of participant data and biological specimens {26b}

Not applicable. This trial does not involve collecting biological specimens.

### Explanation for the choice of comparators {6b}

Participants who complete *Media Aware* will be compared to two other groups in the study. First, the *Media Aware* group will be compared to a delayed intervention control group to rigorously test the short- and long-term efficacy of the program. Next, they will be compared to an active control group that receives sexual health programming without MLE content because this will provide information about the unique effects of MLE-based sexual health programming (compared to sexual health programming without MLE) on proposed mediators and outcomes.

### Interventions {11a}

#### Intervention—*Media Aware for Young Adults* (i.e., *Media Aware*)

Students from community college campuses that are randomized to this arm will receive *Media Aware* between the pretest and posttest questionnaire. *Media Aware* is an asynchronous, multimedia, web-based eLearning program that was designed to promote sexual health and develop critical thinking skills through teaching media literacy skills and using media literacy education (MLE) methods. The program is interactive, inclusive, and developmentally appropriate for young adults. *Media Aware* is different from traditional sexual health education programs because students are educated to become more aware of a less conscious influence on sexual behavior choices (i.e., media messages) and develop critical thinking skills to analyze and evaluate media messages. *Media Aware* is a 5-module, comprehensive sexual health MLE program designed to be completed individually on a computer or mobile device and includes a wide range of sexual health topics including pregnancy and STI prevention, dating violence, and relationship health. The program can be completed in one or more sittings and, on average, takes about 2 h to complete. The program is hosted in eTrove, a web-based Learning Management System (LMS).

#### Active control—*Health Aware for Young Adults* (i.e., *Health Aware*)

The active control program (i.e., *Health Aware*) consists of five highly interactive, asynchronous, web-based, eLearning modules that include the same health content (e.g., pregnancy and STI prevention, dating violence, and relationship health), functionality, and interactivity as *Media Aware*, but without the MLE content. All of the videos, activities, and content included in *Media Aware* that *do not* include MLE content are included in *Health Aware*. MLE content includes any activities/instructions designed to impact media-related cognitions or media deconstruction skills; this includes any content about media use, discussion of information contained in or missing from media messages, reflection about media messages, and instruction on critical thinking about media (e.g., media deconstruction activities). This software program is hosted in eTrove, as well.

### Criteria for discontinuing or modifying allocated interventions {11b}

Not applicable. There are minimal risks associated with participation in this study. If a participant experiences any discomfort, they can end their participation immediately and at any time.

### Strategies to improve adherence to interventions {11c}

Use of *Media Aware* and *Health Aware* will be monitored in an LMS. To improve adherence, automated reminders and text messages will be sent to participants if participants are not completing the intervention programs during the time provided. In addition to tracking adherence, several strategies were used during the program development process to create a program that would engage and retain the target audience. First, *Media Aware* was developed using an iterative process such that feedback from the target population was incorporated at multiple time points to enhance its relevance to and acceptance by young adults [10]. *Health Aware* was created from *Media Aware* and, thus, also integrates the input and feedback obtained from use of this iterative design process. Second, because web-based programs are highly acceptable to students, who often report preferring digital learning to other formats [15–17], these sexual health education programs were designed to be completed online using responsive design, so that students can easily access programs across devices including a computer, smartphone, or tablet.

### Relevant concomitant care permitted or prohibited during the trial {11d}

The active control group and delayed intervention control group will not have access to *Media Aware* during the first 12 months of the study. Additionally, only the active control group will have access to *Health Aware*. There are no restrictions, however, on the behavior of participants during the trial.

### Provisions for post-trial care {30}

The delayed intervention control group will receive access to *Media Aware* after completing the 12-month follow-up questionnaire.

### Outcomes {12}

All primary and secondary outcomes will be assessed using online questionnaires at four timepoints: pretest, posttest, 6-month follow-up, and 12-month follow-up. Thus, while controlling for pretest scores, we will evaluate differences across groups in each of our outcomes at posttest, 6-month follow-up, and 12-month follow-up. Continuous variables measured using multiple items will be aggregated by creating scale scores based on mean values. All primary and secondary health outcomes are clinically relevant, as they are related to overall health and well-being, including STI/HIV transmission, unplanned pregnancy, relationship satisfaction, and experiences of sexual violence [18, 19]. Primary health outcomes will include relationship satisfaction, relationship violence perpetration, relationship violence victimization, identity abuse, risky sexual behaviors, condom use at last sex (anal, vaginal), condom and/or dental dam use at last oral sex, contraceptive use at last vaginal sex, and frequency of use of protection during sex (anal, vaginal, oral).

Secondary health outcomes related to relationship health and sexual violence prevention will include normative beliefs related to dating violence and gender roles, acceptance of rape myths, and efficacy and intentions to intervene as a bystander. Secondary health outcomes related to sexual health include attitudes, normative beliefs, self-efficacy, behavioral intentions, and willingness related to (1) risky sexual behaviors; (2) unprotected sex; (3) contraception/protection; and (4) sexual health communication.

Secondary outcomes related to media-related cognitions and behaviors assess whether participants have the skills necessary to critically analyze media messages—skills which are related to health decisions and behaviors [20]. These include perceived media message completeness, cognitive elaboration, perceived realism of media messages, perceived similarity to media messages, identification with media messages, media skepticism, and media deconstruction skills.

### Participant timeline {13}

**Fig. 1:**
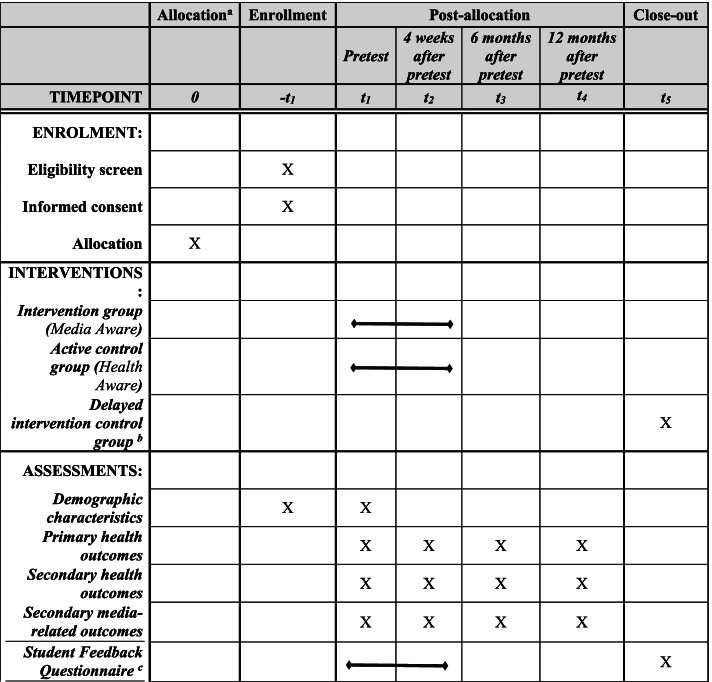
SPIRIT participant timeline. Note: ^a^ Randomization to groups (i.e., intervention, active control, or delayed intervention control) will occur at the campus-level prior to participant enrollment. ^b^ Participants who are in the delayed intervention control group will receive Media Aware after they complete the 12-month posttest. ^c^ Each participant will only receive the student feedback questionnaire once. Participants in the intervention group and active control group will receive the student feedback questionnaire between pretest and posttest (after they complete their assigned program). Participants in the delayed intervention control group will complete the student feedback questionnaire after they receive *Media Aware* (after t4)

Upon enrollment, participants will receive an email and/or text indicating that they have been enrolled in the study, and provided with a link to access the pretest questionnaire (which will use a unique identifier in place of names, for confidentiality). Upon completion of the pretest questionnaire, participants will receive a second email. The content of the second email will depend upon the participants’ condition. Participants on campuses assigned to the intervention group will received an email link to the *Media Aware* program. Participants on campuses in the active control group will receive an email containing a link to the *Health Aware* program. Both of these groups will be told they will have 2 weeks to complete the program and complete the Student Feedback Questionnaire (SFQ). The SFQ is administered following completion of the assigned program (see “Measures” section for more information about the SFQ). Participants on campuses assigned to the delayed intervention control group will receive an email stating that the next step will be the completion of a second questionnaire in 4 weeks (the posttest). Email links for each questionnaire will be sent to all participants at the appropriate times, based upon the completion date of the pretest questionnaire: the posttest questionnaire will be administered 4 weeks after the pretest; the first follow-up questionnaire will be administered 6 months after the pretest; the second follow-up questionnaire will be administered 12 months after the pretest. After the delayed intervention control participants complete the 12-month follow-up questionnaire, they will receive an email that will include a link to access the *Media Aware* program, and they will be asked to complete the program and SFQ. This participant timeline is also shown in Fig. 1.

### Sample size {14}

We expect to recruit approximately 2010 young adults who are attending community college to participate in this study. We plan to recruit approximately 67 students on each of 30 different community college campuses as participants. Optimal Design [21] was used to determine that there would need to be 40 participating students from each college in the final sample for the proposed group randomized trial. Based on a previous evaluation of *Media Aware* [10], the following values were input to determine the final targeted sample size: (1) a small effect size (.28); (2) ICC for campus effects = .02; (3) *α* = .05; (4) power = .80; and (5) 10 campuses per condition. The effect size used in this power analysis was the smallest effect size detected in a previous evaluation of Media Aware with community college students (for one of the secondary outcomes: willingness to have unprotected sex). The ICC used in this power analysis is the one detected in the previous evaluation. With an estimated attrition rate of 40% between pretest and 12-month follow-up, it was determined that 2010 students were needed to complete the pretest in order to achieve the final sample size. However, we intend to multiply impute missing data to retain our original sample size. Finally, while it is our goal to recruit 67 students from each of 30 campuses, there are practical considerations related to the community-based nature of this study (e.g., social distancing policies, limited number of eligible students on a campus, campus dropout) that may impact our final number of colleges and size of our student sample.

### Recruitment {15}

Prior to beginning student recruitment, project researchers will meet with personnel (i.e., administrators, faculty, or staff) at community colleges across the USA to discuss the project and its requirements. Community college campuses that agree to make the opportunity to participate available to their students will be included in the study. Efforts will be made to ensure the campuses that are included are diverse with respect to rurality and the racial-ethnic composition of the student body.

It is expected that we will conduct a rolling enrollment of participants over the course of a 2-year recruitment period. Students will be recruited through use of a variety of print and digital methods. During student recruitment, college personnel may also be asked to assist the research team with disseminating study recruitment materials to students. Individualized recruitment plans will be made for use on each college campus—these will often be based upon suggestions from campus administrators. Some methods that may be used include distribution of digital recruitment materials via emails, listservs, or college-specific courseware such as Blackboard, or social media [22]. Print recruitment materials, such as flyers, posters, and tent-cards, may be posted around campus and distributed to students at tabling events. Recruitment materials will direct students to the study website, where they can learn more about the study, find study team contact information, and complete an online screener. If a student passes the screener, they will be directed to an online study consent form.

Not all eligible students who complete the consent form will be enrolled in the study. To ensure a diverse sample with respect to race-ethnicity, we will attempt to sample students from each campus within strata, so that the composition of the study sample on each campus might reflect the composition of the campus race-ethnicity demographic characteristics. Thus, our goal is to recruit a final sample for each campus that is composed of approximately the same percentage of minority students that are enrolled in the college as well as approximately an equal number of male and female students (acknowledging that a proportion of the sample will identify as gender non-binary and be eligible for participation).

### Assignment of interventions: allocation

#### Sequence generation {16a}

To avoid contamination effects, randomization will occur at the college level. Using a stratified sample, community college campuses will be randomized by the statistician on the project using R’s randomization function to either the (1) intervention group (i.e., *Media Aware*); (2) active control group (i.e., *Health Aware*); or (3) delayed intervention control group.

In addition to random assignment of colleges to conditions, we will attempt to assigned colleges based upon their classification within two strata. First, colleges will be stratified based upon the racial-ethnic composition of the student body. Colleges will be classified as either “Lower minority student body enrollment” (i.e., less than 50% minority enrollment) or “Higher minority student body enrollment” (i.e., at least 50% minority enrollment), based upon data from the National Center for Education Statistics [23]. Second, colleges will be classified by rurality (rural vs. not rural) based upon data from the National Center for Education Statistics [23]. The National Center for Education Statistics categorizes colleges as “Rural,” “Town,” “Suburban,” or “City.” For the current study, we will classify colleges as “Rural,” if the college was categorized as “Rural” by the National Center for Education Statistics. We will classify colleges as “Not Rural,” if the college was categorized as “Town,” “Suburban,” or “City” by the National Center for Education Statistics. We will attempt to stratify schools by these criteria to obtain representation of students within each stratum for each of the study conditions.

#### Concealment mechanism {16b}

The condition allocation will be concealed by the online system which will deliver all study materials (e.g., surveys, online programs).

#### Implementation {16c}

The allocation sequence will be generated by the project statistician, who will not be involved in the recruitment or enrollment of students. The allocation sequence will be used to determine which colleges are in each of the three conditions. The online system will assign each student to the appropriate intervention, based upon the condition assignment of their college campus. Participants will be enrolled through the online system by the project director, after their eligibility is confirmed.

### Assignment of interventions: blinding

#### Who will be blinded {17a}

Assignment will be masked from the student participants, who will not know their group assignment (i.e., intervention vs. active control vs. delayed intervention control). In addition, enrollment will be conducted through the online system and links to all surveys and interventions will be sent via email by our automated online system, rather than being delivered by research team members, to ensure that researcher bias is not introduced at this stage of the study.

#### Procedure for unmasking if needed {17b}

The authors do not anticipate any need for unmasking.

### Data collection and management

#### Plans for assessment and collection of outcomes {18a}

All participants will be emailed and/or texted links to complete web-based surveys at pretest, posttest, 6-month follow-up, and 12-month follow-up. Each survey will include all measures for primary and secondary outcomes, described in the “Measures” section below.

#### Measures

Many of the measures proposed for use in this study were used previously in an evaluation of *Media Aware* with community college students and demonstrated good reliability and validity [10]. The measures were specifically designed to be inclusive of LGBTQ+ participants (e.g., sexual activity was defined on the questionnaire to include oral, anal, and/or vaginal sex). Based on the assessment timepoint, minor wording changes are made to some measures to define the reporting time period for a behavior (e.g., frequency of condom use in the past 1 month vs. past 6 months). Measures assessing relationship satisfaction, identity abuse, and relationship violence victimization and perpetration will only be asked of participants who indicate they are currently in a relationship. Measures assessing frequency of communication about sex with a sexual partner, as well as protection and contraception use behaviors, will only be asked of participants who indicate they have had sex.

##### Primary health outcome measures

We will include the following primary health outcome measures:

1. *Relationship satisfaction.* A 7-item scale will be used to assess participants’ satisfaction with their current romantic relationship [24]. Items such as, “How well does your partner meet your needs?” will be rated on a 5-point Likert-type scale from 1 = *poorly* to 5 = *extremely well*, with higher scores indicating greater relationship satisfaction.
2. *Relationship violence victimization.* To assess relationship violence victimization, participants will be asked to select how often their current or ex-dating partner perpetrated certain acts of violence against them (e.g., “my partner threatened to hurt me”). Ten items [25, 26] will be used to measure experiences of three dimensions of relationship violence victimization: (1) Physical Abuse (assessed with items such as “My partner kicked, hit, or punched me”); (2) Psychological Abuse (assessed with items such as “My partner insulted me with put-downs”); and (3) Sexual Abuse (assessed with items such as “My partner forced me to have sex when I didn’t want to”). Each item will be rated on a 4-point Likert-type scale from 1 = *never* to 4 = *often*.
3. *Relationship violence perpetration.* Ten items [25, 26] will be used to assess experiences of three dimensions of relationship violence perpetration. These items will be parallel to the ones used to assess relationship violence victimization (described above). The only difference between the victimization and perpetration scales is that the perpetration scale assesses examples of relationship violence perpetrated by the respondent.
4. *Identity abuse.* A 7-item measure will be used to assess Identity Abuse [27], which is a distinct form of abuse experienced by the LGBTQ+ community that is correlated with, but distinct from, other forms of intimate partner violence. Only participants who indicate they are currently in a relationship and they are not heterosexual will respond to these items. Participants will be asked to rate how often certain acts of identity abuse (e.g., “The person used my sexual orientation or gender identity against me”) happened on a 7-point Likert-type scale from 0 = *never* to 6 = *more than 20 times*, with higher scores indicating more frequent identity abuse.
5. *Risky sexual behaviors.* Risky sexual behaviors will be evaluated with 4-items which assess the number of times the participant: (1) engaged in sexual activity with a casual sexual partner; (2) engaged in sexual activity with someone with an unknown STI status; (3) engaged in sexual activity with someone who has had many partners; (4) used alcohol or drugs before or during a hook-up or sexual encounter; and (5) engaged in sexual activity with someone engaging in sexual activity with others in the same period [10].
6. *Use of protection as last oral sex.* One item will be used to assess whether participants used protection: “Did you use a condom and/or dental dam the last time you had oral sex?” Response options are “yes” or “no.”
7. *Frequency of use of protection during oral sex.* Three items will be used to assess frequency of use of protection during oral sex [e.g., “In the past month, how often did you or your partner(s) use a condom or dental dam when having oral sex?”]. Items will be rated on a 4-point Likert-type scale from 1 = *never* to 4 = *always*, with higher scores indicating more frequent use of protection.
8. *Frequency of condom use during vaginal sex.* Three items will be used to assess frequency of condom use during vaginal sex [e.g., “In the past month, how often did you or your partner(s) use a condom when having vaginal sex?”]. Items will be rated on a 4-point Likert-type scale from 1 = *never* to 4 = *always*, with higher scores indicating more frequent condom use.
9. *Frequency of birth control use.* Three items will be used to assess frequency of birth control use [e.g., “In the past month, how often did you or your partner(s) use one of the following forms of birth control: Birth control pills, The Shot (DepoProvera), The Patch, The Ring (Nuvaring), IUD (Mirena, Paraguard, Skyla), The Implant (Implanon, Nexplanon), or other FDA approved methods?”]. Items will be rated on a 4-point Likert-type scale from 1 = *never* to 4 = *always* with higher scores indicating more frequent birth control use.
10. *Contraceptive use at last vaginal sex.* One item (i.e., “Did you use any contraceptive method the last time you had vaginal sex?”) will be used to assess contraceptive use at last vaginal sex. Participants will answer “yes” or “no.”
11. *Frequency of condom use during anal sex.* Three items will be used to assess frequency of condom use during anal sex [e.g., “In the past month, how often did you or your partner(s) use a condom when having anal sex?”]. Items will be rated on a 4-point Likert-type scale from 1 = *never* to 4 = *always* with higher scores indicating more frequent condom use.
12. *Condom use at last anal sex.* One item (i.e., “Did you use a condom during your last anal intercourse?”) will be used to assess condom use the last time the participant had anal sex. Participants will answer either “yes” or “no.”

##### Secondary health outcome measures

We will include the following secondary health outcome measures:

1. *Sexual health knowledge.* Knowledge of sexual risk and sexual protection will be assessed through a series of 16 fact-based questions about the transmission of STIs, ways to reduce the risk of STIs, and the effectiveness of contraception/protective methods (e.g., “True or False: You can tell if someone has an STI by looking at him/her”) [10]. For most questions, participants who answer correctly will receive a “1” and participants who answer incorrectly will receive a “0”; however, some questions are worth more than one point (i.e., “choose all that apply” questions). Therefore, once the items are summed, scores will range from 0 to 23 with higher scores indicative of greater sexual health knowledge.
2. *Attitudes toward unprotected sex.* One item [i.e., “It is okay to…have unprotected sex (not including when people are trying to get pregnant)”] will be rated on a 4-point Likert-type scale from 1 = *strongly disagree* to 4 = *strongly agree* to assess attitudes toward unprotected sex. Higher scores will indicate more positive attitudes toward unprotected sex.
3. *Attitudes toward risky sexual behaviors.* A 5-item measure will be used to assess attitudes toward risky sex [28]. Items such as, “It is okay to have sex with someone who has had many sexual partners,” will be rated on a 4-point Likert-type scale from 1 = *strongly disagree* to 4 = *strongly agree*. Higher scores will indicate more positive attitudes toward risky sexual behaviors.
4. *Attitudes toward contraception/protection.* A 9-item measure will be used to assess attitudes toward contraception/protection [28]. Items such as, “It is wrong to use birth control,” will be rated on a 4-point Likert-type scale from 1 = *strongly disagree* to 4 = *strongly agree*. Higher scores will indicate more favorable attitudes toward contraception.
5. *Attitudes toward communication with partners and medical professionals about sex.* A 4-item measure will be used to assess attitudes toward communication with partners and medical professionals [10]. Items such as, “Before deciding to have sex, people should talk with their partner about HIV/AIDS and other STIs,” will be rated on a 4-point Likert-type scale from 1 = *strongly disagree* to 4 = *strongly agree*. Higher scores will indicate more positive attitudes toward communication.
6. *Sex refusal self-efficacy.* A 5-item measure will be used to assess participants’ self-efficacy to refuse sex [29]. Items such as, “I can easily say ‘no’ to someone who is pressuring me to have sex,” will be rated on a 4-point Likert-type scale from 1 = *strongly disagree* to 4 = *strongly agree*. Higher scores will indicate greater self-efficacy.
7. *Self-efficacy to refuse unprotected sex.* Self-efficacy to refuse unprotected sex will be assessed with one item (“I can easily say ‘no’ to sex if we do not have protection even if I really want to have sex with that person”), rated on a 4-point Likert-type scale from 1 = *strongly disagree* to 4 = *strongly agree*. Higher scores will indicate greater self-efficacy.
8. *Self-efficacy to use protection.* Two items (e.g., “I can use a condom correctly or explain to my partner how to use a condom correctly”), will be rated on a 4-point Likert-type scale from 1 = *strongly disagree* to 4 = *strongly agree*, to assess participants’ self-efficacy to use protection when they have sex [29]. Higher scores will indicate greater self-efficacy.
9. *Self-efficacy to communicate with partners and medical professionals about sex*. A 4-item measure will be used to assess self-efficacy to communicate with sexual partners and medical professionals about sex [29]. Items such as, “I can discuss preventing STIs with my partner,” will be rated on a 4-point Likert-type scale from 1 = *strongly disagree* to 4 = *strongly agree*. Higher scores will indicate greater self-efficacy to communicate.
10. *Efficacy to intervene as a bystander.* Five items (e.g., “I could talk to a friend who I suspected is in an abusive relationship”) will assess efficacy to intervene as a bystander to abusive relationships or non-consensual sexual activity [30]. Participants will rate their confidence that they could do the action on a scale from 0 to 100 with higher scores indicative of greater bystander efficacy.
11. *Intentions to have unprotected sex.* One item (i.e., “In the next 6 months, how likely is it that you will have unprotected sex?”) will be rated on a 4-point Likert-type scale from 1 = *not at all likely* to 4 = *extremely likely*. Higher scores will indicate greater intentions to have unprotected sex.
12. *Intentions to use protection/contraception.* A 3-item measure will be used to assess intentions to use condoms and contraception. Items such as, “If you were to decide to have sexual intercourse in the next 6 months, how likely would you be to use a condom?” will be rated on a 4-point Likert-type scale from 1 = *not at all likely* to 4 = *extremely likely* with higher scores indicative of greater intentions to use protection/contraception.
13. *Intentions to communicate with partners and medical professionals about sex.* A 6-item measure will be used to assess intentions to communicate with sexual partners and medical professionals about sex. Items such as, “If you were to decide to engage in sexual activity with a new partner in the next 6 months, how likely would you be to talk with a partner about HIV/AIDS or other STIs?” and “If you were to decide to engage in sexual activity with a new partner in the next 6 months, how likely would you be to talk with a doctor or other medical professional beforehand?,” will be rated on a 4-point Likert-type scale from 1 = *not at all likely* to 4 = *extremely likely*. Higher scores will indicate greater intentions to communicate.
14. *Risky sexual behavior intentions.* A 5-item measure will be used to assess intentions to engage in risky sexual behaviors. Items such as, “In the next 6 months, how likely is it that you will have oral, anal, or vaginal sex with a casual partner?” will be rated on a 4-point Likert-type scale from 1 = *not at all likely* to 4 = *extremely likely* with higher scores indicative of greater intentions to engage in risky sex.
15. *Intentions to intervene as a bystander.* A 4-item scale will be used to assess intentions to intervene as a bystander to abusive relationships or non-consensual sexual activity [31]. The question stem will prompt participants to rate the likelihood (on a 4-point Likert-type scale from 1 = *not at all likely* to 4 = *extremely likely*) that they would engage in four behaviors, including: “Approach a friend if I thought they were in an abusive relationship and let them know that I am here to help.” Higher scores will indicate greater intentions to intervene as a bystander.
16. *Willingness to have unprotected sex.* One item will be used to assess willingness to have unprotected sex [32]: “Suppose you were with your boyfriend/girlfriend/partner. He/she wants to have sex, but neither of you have any form of protection. In this situation, how willing would you be to go ahead and have sex anyway?” The item will be rated on a 4-point Likert-type scale from 1 = *very unwilling* to 4 = *very willing*. Higher scores will indicate greater willingness to have unprotected sex.
17. *Willingness to engage in risky sexual behaviors.* A 5-item measure will be used to assess willingness to engage in risky sexual behaviors, with items such as [32]: “Suppose you wanted to have sex with someone but you did not know their STI status. In this situation, how willing would you be to have sex anyway?” Items will be rated on a 4-point Likert-type scale from 1 = *very unwilling* to 4 = *very willing* with higher scores indicative of greater willingness to have risky sex.
18. *Descriptive norms of unprotected sex.* One item will be used to assess descriptive norms of unprotected sex: “What percentage of people your age have had unprotected sex?” Participants will write in their estimate of what percentage of their peers (i.e., 0–100%) are engaging in the behavior. Higher scores will indicate participants think more of their peers are engaging in unprotected sex.
19. *Descriptive norms of risky sexual activity.* A 5-item measure will be used to assess descriptive norms of peer engagement in risky sexual activity. Participants will write in their estimate of what percentage of their peers (i.e., 0–100%) are engaging in risky sexual behaviors, such as, “oral, anal, or vaginal sex with someone who has not been tested for STIs or whose STI status is unknown.” Higher scores will indicate participants think more of their peers are engaging in risky sex.
20. *Descriptive norms of similar peers’ risky sexual activity.* One item will be used to assess descriptive norms of similar peers’ engagement in risky sexual behaviors: “Most people like me use protection when they have sex.” The item will be rated on a 4-point Likert-type scale from 1 = *strongly disagree* to 4 = *strongly agree* with higher scores indicative of less risky descriptive norms.
21. *Injunctive norm of “most people” about risky sex.* A 3-item measure will be used to assess whether participants believe that most people think that it is okay for people their age (i.e., the participants’ age) to engage in risky sexual behaviors. Items such as, “Most people believe that it is okay for people my age to have unprotected sex,” will be rated on a 4-point Likert-type scale from 1 = *strongly disagree* to 4 = *strongly agree*. Higher scores will indicate the participant has riskier injunctive norms.
22. *Injunctive norms of “friends” about risky sex.* Two items will be used to assess whether participants believe that their friends think that it is okay for them to engage in risky sexual behaviors. Items such as, “My friends think I should use protection when I have sex,” will be rated on a 4-point Likert-type scale from 1 = *strongly disagree* to 4 = *strongly agree*. Higher scores will indicate the participant has less risky injunctive norms.
23. *Gender role norms.* A 6-item measure will be used to assess participants’ acceptance of traditional gender norms [33]. Items such as, “Raising children is primarily a woman’s responsibility,” will be rated on a 4-point Likert-type scale from 1 = *strongly disagree* to 4 = *strongly agree*. Higher scores will indicate less acceptance of nontraditional gender roles.
24. *Dating violence norms.* A 4-item measure will be used to assess participants’ dating violence norms [33]. Items such as, “It is OK for people to hit their girlfriends/boyfriends/partners, if they did something to make them mad,” will be rated on a 4-point Likert-type scale from 1 = *strongly disagree* to 4 = *strongly agree*. Higher scores will indicate more acceptance of dating violence.
25. *Rape myth acceptance.* Three subscales (“she asked for it,” “it wasn’t really rape,” and “he didn’t mean to”), with a total of 13 items, from the Illinois Rape Myth Acceptance – Short Form will be used to assess participants’ attitudes toward rape myths [34]. Items such as, “If a girl is raped while she is drunk, she is at least somewhat responsible for letting things get out of hand,” will be rated on a scale from 1 = *strongly disagree* to 4 = *strongly agree*. Higher scores will indicate greater rape myth acceptance.
26. *Frequency of communication about sex with sexual partners.* Six items will be used to assess frequency of sexual communication with a partner(s) [e.g., “In the past month, how often did you talk to your partner(s) about sexually transmitted infections (STIs)?”]. Items will be rated on a 4-point Likert-type scale from 1 = *never* to 4 = *always*. Higher scores will indicate more frequent communication.
27. *Communication with a doctor about sex.* Three items will be used to assess whether participants communicated with a doctor about sex (e.g., “In the past month, had you talked to a doctor or other medical professional about sex, contraception, and/or relationships?”). Participants will answer “yes” or “no.”

##### Secondary media-related outcomes measures

We will include the following media-related outcome measures:

1. *Perceived realism of media.* A 6-item measure will be used to assess the degree to which participants believe media portrayals are similar to real-life people and events [35]. Items such as, “People my age in the media have sexual contact as often as average people my age,” will be rated on a 4-point Likert-type scale from 1 = *strongly disagree* to 4 = *strongly agree*. Higher scores will indicate participants think media are more realistic.
2. *Perceived similarity to media.* A 7-item measure will be used to assess whether participants believe media portrayals are similar to their personal experiences [35]. Items such as, “The things I do in my life are similar to what I see in the media,” will be rated on a 4-point Likert-type scale from 1 = *strongly disagree* to 4 = *strongly agree*. Higher scores will indicate greater perceived similarity*.*
3. *Identification with media.* A 3-item scale will be used to assess the extent to which participants identify with the people and behaviors they see in media messages [35]. Items such as, “I want to do the things that people my age in the media do,” will be rated on a 4-point Likert-type scale from 1 = *strongly disagree* to 4 = *strongly agree*. Higher scores will indicate participants identify more with media.
4. *Media skepticism.* A 6-item measure will assess the degree to which participants disbelieve or discount media messages [10]. Items such as, “The media are dishonest about what happens when people drink alcohol,” will be rated on a 4-point Likert-type scale from 1 = *strongly disagree* to 4 = *strongly agree*. Higher scores will indicate participants have more media skepticism.
5. *Media deconstruction skills.* A performance measure with open-ended questions will be used to assess media deconstruction skills. To start, participants will view an advertisement which uses themes related to sexuality and romantic relationships to promote alcohol consumption [13]. Next, participants will be asked to: “Tell us about the advertisement in the space below (the more detail the better).” They will also be asked to respond to the following: “How are advertisers trying to get someone to buy this product?” Qualitative responses to the questions will be coded by trained project staff members once inter-coder reliability is established, and scores will be summed to create an overall deconstruction skills composite variable.
6. *Perceived media message completeness.* One item will assess perceived media message completeness: “How complete is the information in this advertisement?” Participants will view an advertisement which uses themes related to sexuality and romantic relationships to promote alcohol consumption [13], and then answer the question on a 4-point Likert-type scale from 1 = *incomplete* to 4 = *complete* [10]. Higher scores will indicate less critical media analysis of the advertisement*.*
7. *Cognitive elaboration.* After participants view an advertisement which uses themes related to sexuality and romantic relationships to promote alcohol consumption [13], they will answer three items to assess the time they spent thinking about the advertisement presented (i.e., cognitive elaboration). Items such as, “How much time did you spend thinking about this advertisement?” will be rated on a 4-point Likert-type scale from 1 = *not much at all* to 4 = *a lot* [36]. Higher scores will indicate greater cognitive elaboration of the advertisement.

##### Student Feedback Questionnaire (SFQ)

A series of Likert-type questions will gather feedback from participants on program satisfaction including program relevance, usability, engagement, inclusivity (e.g., gender, race, ethnicity, and sexual orientation), potential for improving the health of community college students, and net promoter score (i.e., likelihood of recommending to other community college students) [37]. Three open-ended questions will allow participants to indicate what they liked best and least about the program, and what they learned in the program. In the SFQ, the intervention and delayed intervention control groups will provide feedback on *Media Aware* and the active control group will provide feedback on *Health Aware*.

##### Other measures

Data on demographic characteristics will be collected, including age, sex, gender, sexual orientation, race, and ethnicity. Participants will be asked to report their student status, year in school, Pell Grant qualification (as a measure of SES), and international student status. They will be asked their current housing situation, relationship status, number of children, whether they are currently trying to get pregnant, and primary source of health insurance. Participants will be asked their religious affiliation (e.g., Protestant, Atheist, Muslim), as well as respond to items to assess their religiosity. Data on process measures will also be collected. Two indicators of dosage can be obtained from the LMS including the amount of time that each learner spends in the program and the amount of progress made in the program. Adherence is assessed by the number of times the user clicks on a unique interactivity in the program (e.g., quiz questions, “click to reveal” activities).

#### Plans to promote participant retention and complete follow-up {18b}

Contact information for participants, including phone numbers, mailing addresses, secondary email addresses (if available), and contact information for a friend or family member will be collected on the consent form to support follow-up efforts, if email addresses receive no response or change during the study. Texts will be sent as another method for keeping in contact with participants. The incentive structure is also expected to support participant retention in this study. Participants will receive a $20 gift card for completing the pretest questionnaire, a $30 gift card for completing the SFQ, a $30 gift card for completing the posttest questionnaire, a $40 gift card for completing the 6-month follow-up questionnaire, and a $50 gift card for completing the 12-month follow-up questionnaire.

#### Data management {19}

Electronic documentation of consent will be stored on a secure server separately from study data. Data will be stored with randomly assigned ID numbers. Questionnaires and program process data will not include names or personally identifying information (PII); thus, there will be no way to directly link individuals’ responses to their names or other identifying information. A separate linking list that will include identities and ID numbers will be kept confidentially on a secure server that is only accessible to project members. After the project has ended and the data have been verified, the linking list will be destroyed rendering the data anonymous. All data collected will only be used for the research purposes approved, in accordance with protocols approved by the IRB. Data collection will be conducted using a secure web-based software system that allows for de-identified data export. A de-identified data set will be exported for data analysis, and any subsequent manuscripts, reports, or presentations will not contain PII. Finally, individual participants will not be identifiable in manuscripts, reports, or presentations about the research.

#### Confidentiality {27}

In the consent forms, prospective participants will be told that their responses and participation in the study will be kept confidential. In addition to the data management protocol described above, there are a number of strategies we will use to maintain participant confidentiality. First, during the onboarding process of new employees, they are required to sign a confidentiality agreement regarding the protection of proprietary information. Second, new employees complete several training courses on the protection of human subjects in research. Third, project team members receive instruction regarding strategies to maintain the anonymity of participants including to never discuss specific participants, even without using their names, in any location where they might be overheard.

#### Plans for collection, laboratory evaluation and storage of biological specimens for genetic or molecular analysis in this trial/future use {33}

Not applicable. No biological specimens will be collected.

### Statistical methods

#### Statistical methods for primary and secondary outcomes {20a}

Preliminary analyses initially will involve data entry and management activities of both qualitative and quantitative data. Project staff member coders will be trained on a previously validated coding protocol on a dataset separate from the data collected in this study until their coding is reliable. Qualitative data will be coded and appropriate sums and frequencies will be calculated, and emergent themes will be identified.

Psychometric analyses will be conducted to study the reliability, validity, and distributions of quantitative measures. Handling of scales or measures with poor reliability or validity will include modifying or eliminating them from the analysis data set. Variables with markedly skewed distributions will be transformed or categorized to reduce the impact of non-normality on subsequent parametric analyses. The impact of random assignment on producing equivalent groups will be assessed on pretest measures. We will undertake analyses to determine if important differences in students’ demographic characteristics and pretest outcome variables emerge between the three conditions. Campuses will be randomly assigned to treatment groups, so it is especially important that we evaluate the effects of this process on the distribution of students to these conditions, who are not themselves randomly assigned. Group equivalence analyses will be conducted using a series of multilevel linear and logistic regression models to determine whether condition assignment is disproportionately associated with background variables or baseline values of outcome variables. Regression models will include a random intercept for campus, to adjust for the nestedness of the sample. Analyses will be conducted by drafting two macros in SAS, one for use with dichotomous outcomes and one for use with continuous outcomes. Demographic characteristics and baseline outcomes that are nonequivalent across groups will be included as covariates in the main analyses. We will use the procedures outlined by Keeble and colleagues [38] to determine if statistical methods (e.g., weighting; sensitivity analyses) are needed to further reduce the potential impact of selection biases.

Missing data will be treated during the preliminary analysis phase. Similar to the group equivalence analyses, a set of preliminary analyses will be conducted to estimate whether background and auxiliary variables from the pretest assessment (e.g., conscientiousness, access to internet) are associated with missing measurement occasions. Dichotomous variables indicating whether or not participants completed each follow-up assessment (i.e., posttest, 6-month follow-up; 12-month follow-up) will be calculated and multilevel models will be estimated to determine whether variables are associated with missingness at each time point. Demographic background variables and any auxiliary variables that are associated with missingness will be included in multiple imputation procedures. Data will be multiply imputed using the PROC MI procedure in SAS, with the number of imputations based on the degree of missingness in the outcome data. Output imputed datasets will be used for primary analyses, and estimates and standard errors will be pooled using PROC MIANALYZE in SAS, adjusting for within- and between-imputation variation, using Rubin’s Rules [39].

##### Statistical approach for continuous outcomes

Outcomes with a continuous distribution will first be assessed via univariate analyses to determine whether their distributions are appropriate for parametric analyses. If the distributions are sufficiently non-normal, then the variables will be dichotomized and assessed using categorical data analyses, or transformed to follow a normal distribution. If outcomes have a normal distribution, then they will be analyzed using a multilevel linear regression framework, with a random intercept at the campus level to adjust for data nestedness of participant within campus. The dependent variable for each analysis will be the outcome at a specific measurement occasion (i.e., posttest, 6-month follow-up, 12-month follow-up) and independent variables will include the pretest level of the outcome, group membership contrast (*Media Aware* vs. active control or *Media Aware* vs. delayed intervention control), and gender. Gender will be included as a covariate in analyses due to previous studies that showed consistent gender differences across many sexual and relationship health outcomes [40–42]. If group equivalence analyses determine that groups are significantly disproportionate in terms of background variables assessed, then those background variables also will be included as covariates in the main analyses. Analyses will be conducted on each imputed data set, and fixed and random effects results will be pooled using Rubin’s Rules [39]. All analyses will be estimated using a SAS macro that will conduct analyses on each data set, pool results together, and output fixed and random effects, and an effect size for the group comparison, in the form of Cohen’s *d*.

##### Statistical approach for dichotomous outcomes

Outcomes with a dichotomous distribution will be analyzed using a multilevel logistic regression framework, with a random intercept at the campus level to adjust for data nestedness of participant within campus. The dependent variable for each analysis will be the dichotomous outcome at a specific measurement occasion (i.e., posttest, 6-month follow-up, 12-month follow-up) and independent variables will include the pretest level of the outcome, group membership contrast (*Media Aware* vs. active control or *Media Aware* vs. delayed intervention control), and gender. If group equivalence analyses determine that groups are significantly disproportionate in terms of background variables assessed, then those background variables will be included as covariates in the main analyses. Analyses will be conducted on each imputed data set and fixed and random effects results will be pooled using Rubin’s Rules [39]. All analyses will be estimated using a SAS macro that will conduct analyses on each data set, pool results together, and output fixed and random effects and an effect size for the group comparison, in the form of an odds ratio.

##### Adjusting for type I error

Due to the large number of outcomes involved in this project, we will employ the Benjamini-Hochberg procedure to avoid detecting false positive results [43]. First, outcomes that are substantively similar will be grouped into “families of outcomes.” Second, *p*-values for the group effect for those outcomes will be rank ordered within the outcome family. Third, Benjamini-Hochberg (BH) critical values will be calculated based on each outcome’s rank within the family, how many outcomes are in the family, and the false discovery rate, which will be set to 0.05 for this project. Fourth, the cutoff value for the BH critical values will be identified for each family of outcomes by comparing *p*-values to BH values. The highest *p*-value that is lower than the BH critical value will serve as the BH cutoff value. All *p*-values that fall under the BH critical value will be considered significant.

#### Interim analyses {21b}

This is a minimal risk study, and we do not anticipate any study-related adverse events. However, if a serious adverse event occurs, it will be evaluated by the investigators and study team and reported to the IRB, Independent Monitor, and NIH Program Officer. Additionally, we will conduct analyses on data collected at posttest and at the 6-month follow-up, prior to the end of the study. If it were determined that harm was being done, the investigators would consult the IRB and NIH Program Officer regarding stopping the trial.

#### Methods for additional analyses (e.g., subgroup analyses) {20b}

##### Moderator analyses

After main analyses are complete, analyses will be re-estimated to determine whether program effectiveness varies based on subpopulation. Three specific subpopulations, defined by binary variables of gender, prior sexual experience, and relationship status will be included as potential moderators. Separate multilevel models will examine each moderator variable in interaction with treatment condition, controlling for pretest variable and other covariates, to determine whether the effectiveness of *Media Aware* depends upon subpopulation.

In addition, analyses will consider whether implementation differences might moderate intervention effectiveness on outcome variables. These analyses will determine how the effectiveness of MLE for sexual health promotion may vary as a function of student and implementation characteristics. Taking into consideration that not all students will be motivated to complete a health education program, complier average causal effect (CACE) modeling will be conducted to identify the impact of non-compliance on program impact.

##### Analysis of underlying mechanisms

To explore the mechanisms underlying *Media Aware*’s effects on health outcomes, a series of multilevel structural equation models (MSEM) will be estimated. The MSEM approach allows for simultaneous estimation of outcomes while accounting for the non-independence of the data structure. The first set of analyses will focus on the potential role of media-related variables as mediators between *Media Aware* and primary and secondary outcomes. Mediation will be examined for both immediate posttest outcomes as well as for the 6-month and 12-month follow-up outcomes. The second set of analyses will evaluate whether the mechanisms of change related to the secondary health outcomes for *Media Aware* and the active control program are different. A multiple group MSEM will be estimated to determine whether the underlying mechanisms (i.e., secondary health outcomes) occurring among those in the *Media Aware* program are identical to those in the active control group. We will first establish factorial invariance before testing group differences to demonstrate that equating factor loadings and intercept variances across groups are tenable constraints, thus making comparison between latent parameters possible. We will create higher level phantom latent variables and equate regression parameters across groups to establish a baseline model and then test group differences (i.e., moderated mediation) by sequentially freeing one parameter at a time and calculating the change in the model fit chi-square. The multiple group MSEM approach allows us to simultaneously estimate multiple outcomes while accounting for the nested nature of the data.

##### Process and satisfaction analyses

*Media Aware* will be used by participants in the intervention group (in between pretest and posttest) and the delayed intervention control group (after their 12-month follow-up). *Health Aware* will be completed by participants in the active control group. Process data obtained from use of the program and feedback data obtained from the SFQ will be examined to provide insights into whether *Media Aware* (an MLE approach to sex education) is more acceptable, feasible, appropriate, and engaging than *Health Aware* (a more traditional approach to sex education). First, the average percentage of content successfully completed will be calculated for each group. An independent samples *t*-test between proportions will be performed to determine whether there is a significant difference in completion rates between the two groups. Second, mean scores and descriptive statistics for each construct (e.g., relevance, engagement) on the SFQ will be calculated. Satisfaction constructs will have a response scale of 1–5. The percentage of participants who were satisfied with each program will be calculated and compared via independent samples *t*-test to determine whether there was a significant difference in the satisfaction rates between the two groups. Third, open-ended feedback will be summarized, paying careful attention to repeated themes across participants; examined for the range and diversity of comments, experiences, and perceptions elicited by participants; and explored to pinpoint parts of the programs that were reported to be exceptional as well as areas in need of attention. Taken together, this open-ended feedback will elucidate what content and features of MLE and traditional sexual health education programs are perceived both positively and negatively by young adults.

#### Methods in analysis to handle protocol non-adherence and any statistical methods to handle missing data {20c}

For each follow-up measurement occasion, a dichotomous variable indicating whether or not a participant completed the follow-up assessment will be created. Then, a set of preliminary multilevel logistic models will be conducted to determine whether background and auxiliary variables from the pretest assessment (e.g., conscientiousness, access to internet) are associated with missing measurement occasions. Demographic background variables, any auxiliary variables that are associated with missingness, and any additional variables that are expected to be part of main analyses will be included in the multiple imputation models. Given the large number of survey items at each assessment occasion, the multiple imputation procedure will be split into multiple sections, because including all of the items in a single imputation model will likely result in non-convergence. Instead, items from similar “outcome families” will be grouped together and multiply imputed. Data will be multiply imputed using the PROC MI procedure in SAS, with the number of imputations (*m*) based on the degree of missingness in the outcome data. There will be one output file for each “outcome family” and the file will contain *m* datasets stacked on top of each other, with a variable indicating the imputation number. All analyses will be conducted *m* times and results will be pooled using the PROC MIANALYZE procedure in SAS, which adjusts for between- and within-imputation variance. Given the outcome scales included in this study, we do not expect to have outlying values that would compromise the imputation procedure.

Complier average causal effect (CACE) models will be used to estimate causal effects in the presence of treatment non-compliance. For each outcome, results will be fit with both CACE and intent-to-treat models. CACE methods model compliance and non-compliance in the control group as a latent variable, but only effects for compliers are reported (treatment effects for non-compliers will be fixed to zero to meet the exclusion restriction assumption). Treatment effects will be estimated by regressing outcomes on a binary predictor representing treatment (1) versus active control (0). We will also include covariates that predict compliance to further protect against the introduction of bias that may violate the exclusion restriction assumption due to some participants completing a partial amount of the treatment.

#### Plans to give access to the full protocol, participant level-data and statistical code {31c}

To ensure data confidentiality for all participants, we will make the data and associated documentation available to users based upon reasonable requests, only under the terms of a data-sharing agreement that provides for (1) a commitment to using the data only for IRB-approved research purposes and not to identify any individual participant; (2) a commitment to securing the data using appropriate computer technology; and (3) a commitment to destroying or returning the data after analyses are completed.

### Oversight and monitoring

#### Composition of the coordinating center and trial steering committee {5d}

All project staff members will meet on a regular basis in order to monitor the protocol, resolve issues regarding the project, and to make sure that participant safeguards are constantly being properly maintained. Questions regarding data collection will be immediately brought to the attention of the PI and the Co-I’s. The project director will monitor the study progress on a day-to day basis and report any questions or issues to the PI and Co-I’s. The team will also hold monthly meetings during which all team members will meet to discuss the project. While there are no Stakeholder and Public Involvement Groups for this trial, the project director will check in regularly (i.e., weekly or monthly—depending on the needs of the site) with contacts at the recruitment sites while recruitment is taking place.

#### Composition of the data monitoring committee, its role and reporting structure {21a}

Dr. Laura Widman is the Independent Monitor on this minimal risk study and will perform an independent review of ongoing study progress and safety. The independent monitor is independent from the sponsor organization and has no competing interests. Progress reports, including participant recruitment, attrition, unanticipated problems, and adverse events, will be provided in aggregate to the Independent Monitor. A summary of the year’s participant recruitment and compliance with enrollment criteria, aggregate status of all enrolled participants, attrition rates and reasons for attrition, and adverse/unexpected events will also be sent to the Independent Monitor. The annual report will include whether there are any conditions present whereby the study might be terminated prematurely. This information will also be included in the annual IRB report.

#### Adverse event reporting and harms {22}

This is a minimal risk study and we do not anticipate any study-related adverse events. However, any adverse events that occur will be evaluated by the investigators and reported to the IRB and Independent Monitor promptly. Serious adverse events will be evaluated within 24 h, and any other anticipated problems or events will be evaluated within 72 h; reporting to the IRB and Independent Monitor will occur within 2 weeks.

#### Frequency and plans for auditing trial conduct {23}

Progress reports will be sent to Dr. Laura Widman, the Independent Monitor on this trial, on a quarterly basis. We will also submit a summary of trial progress—including details related to enrollment, attrition, and any adverse/unexpected events—to both the independent monitor and National Institutes of Health. Finally, we have received IRB approval and will submit for annual renewal of this approval throughout the period during which we are recruiting and collecting data for the project.

#### Plans for communicating important protocol amendments to relevant parties (e.g., trial participants, ethical committees) {25}

Important protocol modifications (e.g., changes to eligibility criteria, outcomes, analyses) will be reported to the IRB and all other relevant parties (which could include the Independent Monitor, the trial registry, the NIH program officer, and others).

#### Dissemination plans {31a}

The protocol and results for this study will be submitted to ClinicalTrials.gov. Furthermore, the research findings generated from this grant award will be presented at scientific conferences and webinars, and published in peer-reviewed scientific journals. Final versions of all accepted peer-reviewed manuscripts that arise from this project will be submitted to the digital publication archive, PubMed Central.

## Discussion

Community college students are in need of effective sexual and relationship health promotion resources. While community college students make up nearly half of all undergraduates in the USA, they are often overlooked by research, which can leave them underserved by health education policies and sexual health resources. *Media Aware* was developed to close this resource gap and promote the sexual and relationship health of young adults through use of MLE. Although the program was found to have promisingly positive impacts on media- and health-related outcomes in a recent short-term evaluation conducted with community college students, a number of critical questions remain—questions which the current study is well-positioned to answer.

This study will be the first to isolate the effects of MLE in sexual health promotion and, thus, determine the unique effects of including MLE in sexual health programming. Relatedly, this study will identify the moderators of MLE’s impact on health outcomes, and explore if and how the impacts of MLE may differ for subpopulations of young adults attending community college. This project has the potential to advance theory in MLE and sexual health promotion which could inform future intervention development and implementation. Additionally, this study aims to replicate and extend the findings from the short-term evaluation of *Media Aware* to examine evidence for long-term behavioral effects. Thus, this study could establish a strong evidence base for the effectiveness of *Media Aware* for use with young adults attending community college and could help identify how to optimize the long-term impact of the program on health behaviors.

## Trial status

This is the first version of the protocol. This study began on July 21, 2021, and recruitment is expected to be completed by September 2023.

## Data Availability

The final de-identified data for this protocol may be supplied on reasonable request. Please see the above section, “Plans to give access to the full protocol, participant level-data and statistical code {31c},” for more information about our protocol for sharing data and other materials.

## Abbreviations

MLE: Media literacy education
STIs: Sexually transmitted infections
SFQ: Student feedback questionnaire
BH: Benjamini-Hochberg
CACE: Complier average causal effect
MSEM: Multilevel structural equation models
LMS: Learning management system
